# NGcGM3 Ganglioside: A Privileged Target for Cancer Vaccines

**DOI:** 10.1155/2010/814397

**Published:** 2010-10-27

**Authors:** Luis E. Fernandez, Mariano R. Gabri, Marcelo D. Guthmann, Roberto E. Gomez, Silvia Gold, Leonardo Fainboim, Daniel E. Gomez, Daniel F. Alonso

**Affiliations:** ^1^Vaccine Department, Center of Molecular Immunology, Havana 11600, Cuba; ^2^Laboratory of Molecular Oncology, Quilmes National University, Roque Saenz Peña 352, Bernal B1876BXD Buenos Aires, Argentina; ^3^Elea Laboratories, C1417AZE Buenos Aires, Argentina; ^4^Chemo-Romikin, C1061ABC Buenos Aires, Argentina; ^5^Division of Immunogenetics, José de San Martín Clinics Hospital, University of Buenos Aires, C1120AAF Buenos Aires, Argentina

## Abstract

Active specific immunotherapy is a promising field in cancer research. N-glycolyl (NGc) gangliosides, and particularly NGcGM3, have received attention as a privileged target for cancer therapy. Many clinical trials have been performed with the anti-NGc-containing gangliosides anti-idiotype monoclonal antibody racotumomab (formerly known as 1E10) and the conjugated NGcGM3/VSSP vaccine for immunotherapy of melanoma, breast, and lung cancer. The present paper examines the role of NGc-gangliosides in tumor biology as well as the available preclinical and clinical data on these vaccine products. A brief discussion on the relevance of prioritization of cancer antigens in vaccine development is also included.

## 1. Ganglioside-Based Cancer Vaccines

The field of cancer vaccines definitively changed after April 2010 when the US Food and Drug Administration (FDA) approved Provenge (sipuleucel-T), a vaccine for advanced prostate cancer patients [1]. For the first time sipuleucel-T convincingly increased overall survival by about four months in a randomized Phase III trial conducted in 512 patients. While this immunotherapeutic agent is relatively inconvenient as a personalized vaccine, it seems that selecting the recombinant version of the prostatic acid phosphatase (PAP)—expressed in 95% of prostatic tumor cells—as antigen was critical [2], reaffirming target selection as a key feature in cancer vaccine design.

Reasoning in the same way, gangliosides, a broad family of structurally related glycolipids, were firstly suggested as potential targets for cancer immunotherapy [3, 4] based on their higher abundance in tumors when compared with the matched normal tissues. Disappointingly, at the beginning of the present century the failure of the best developed ganglioside-based cancer vaccine for that time, GMK [5], irradiated an unfavorable risky perception to all the projects in the field, even those not related with the target antigen, the GM2 ganglioside.

Nevertheless, in these days ganglioside cancer vaccinologists were facing a kind of “polarization” of positions in view of new facts. Eggermont et al. reported an earlier stop of the Phase III GMK vaccine clinical trial in stage II melanoma patients because the Independent Data Monitoring Committee detected an inferior survival rate for the vaccine arm [6]. While difficult to interpret with respect to potential detrimental effects, this result was considered as a significant setback for ganglioside-based specific immunotherapy in melanoma. In the opposite side, a more optimistic outlook of gangliosides as targets for active immunotherapy of cancer came from the recent work by Cheever et al., who reported in 2009 the National Cancer Institute pilot project for prioritization of cancer antigens [7]. From the selected 75 representative antigens, 4 were gangliosides (GD2, GD3, fucosyl-GM1, and N-acetyl-GM3), ranking between positions 12 and 48.

As an overall, at the present moment only two N-glycolyl (NGc) ganglioside-based vaccines, the focus of this paper, are currently tested in Phase III clinical trials [8]. These include racotumomab (formerly known as 1E10) and NGcGM3/VSSP vaccine (Table 1). Racotumomab is an anti-idiotype murine monoclonal antibody (mAb) to NGc-containing gangliosides. An anti-idiotype mAb, such as racotumomab, is the mirror image of the original antibody formed against specific surface antigens. Thus, anti-idiotype antibodies can act as antigens, inducing a response against the original antigen. On the other hand, the NGcGM3/VSSP vaccine results from the conjugation of the ganglioside into very small size proteoliposomes (VSSP) derived from *N. meningitidis*.

## 2. NGc Gangliosides in Tumor Biology

The most common sialic acids in mammals are N-acetylneuraminic acid and N-glycolylneuraminic acid, usually found as terminal constituents of different membrane glycoconjugates such as the GM3 ganglioside. The only structural difference between them consists of a single oxygen atom at the C-5 position of N-glycolylneuraminic acid, catalyzed by the cytidine monophospho-N-acetylneuraminic acid hydroxylase (CMAH) [9]. In contrast to most mammals, including our closest relatives: the great apes, NGc is practically undetectable in healthy human tissues and fluids [10], since human cells lack the presence of CMAH [11]. It is known that this absence is due to the loss of a 92-bp segment in the exon 6, which results in a frameshift mutation of the human gene by an Alu-mediated inactivation [12, 13] dated 2.5–3 million years ago, prior to brain expansion during human evolution [14, 15].

**Figure 1:**
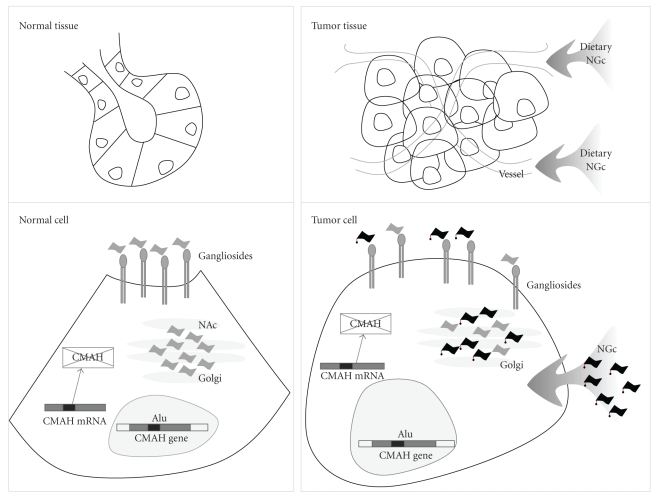
NGc gangliosides in human tumor biology. Although NGcGM3 is practically undetectable in healthy human tissues as a result of an Alu-mediated inactivation of the gene, the ganglioside is highly expressed in several human cancer cells presumably due to incorporation of dietary NGc.

It is noteworthy that the monosialic acid ganglioside NGcGM3 is highly expressed in several human cancer cells [16]. Although initially it was suggested that NGc could be expressed in human tissues by an alternative metabolic pathway [17], nowadays plenty of evidence suggests that the presence of this sialic acid in human cancer is the result of the metabolic incorporation of dietary NGc [18, 19], as illustrated in Figure 1. We reported that cultured mouse tumor cells lacking CMAH expression are able to process and incorporate NGc from different sources such as bovine serum, NGc-rich mucins, or purified N-glycolylneuraminic acid, thus, promoting the metastatic phenotype [20]. Moreover, genetically modified mice expressing a human-like CMAH mutation showed no endogenous NGc, as in humans [21].

The significance of NGc overexpression in human cancer is still under investigation. Taking into consideration that an anti-NGc antibody response was detected in several cancer patients, Varki [22] recently hypothesized that antibody-mediated inflammation could facilitate tumor progression. However, it is accepted that high titers of these antibodies can kill tumor cells [22]. In addition, experimental results obtained by de León et al. indicated that growth-stimulating features of the NGc on tumor cells can be explained by immune system down modulation [23].

## 3. Preclinical Data

Our team analyzed the antitumor activity and the preclinical toxicity of the mAb racotumomab and the NGcGM3/VSSP vaccine using different animal models. Considering that the most important feature of an anti-idiotype mAb (Ab2) is its biological effect, racotumomab was evaluated in two syngeneic murine tumor models, the F3II mammary carcinoma (BALB/c mice) and B16 melanoma (C57BL/6 mice). Both cell lines are positive for the idiotype mAb P3 (Ab1), which specifically reacts with NGc-containing gangliosides on cell surface [24, 25]. In BALB/c mice, vaccination with several intraperitoneal doses, at 14-day intervals of racotumomab coupled to keyhole limpet hemocyanin in Freund's adjuvant, significantly reduced subcutaneous tumor growth of F3II mammary carcinoma cells and the formation of spontaneous lung metastases [24]. Similarly, intravenous administration of uncoupled racotumomab, as a biological response modifier, dramatically inhibited metastatic lung colonization by B16 melanoma cells in C57BL/6 mice [24].

Vaccination with aluminum hydroxide-precipitated racotumomab induced antimetastatic effects in the 3LL-D122 Lewis lung carcinoma, a poorly immunogenic and highly metastatic model in C57BL/6 mice [37]. The effect was associated to T cell infiltration, enhancement of tumor apoptosis, and reduction of new blood vessels formation in lung nodules. The 3LL-D122 lung carcinoma is an antigen-positive, validated model for the NGcGM3 ganglioside. The model evidenced an increased expression of such specific antigen from primary tumors to metastatic lesions [38]. Immunization with the NGcGM3/VSSP vaccine, prepared either with synthetic or natural source-derived ganglioside, showed similar immunogenicity profiles and antitumor effects in the 3LL-D122 model [38].

Racotumomab also demonstrated a potent antitumor effect in combination with chemotherapy in preclinical studies, providing a rationale for chemo-immunotherapy combinations in solid cancers. Administration of low-dose cyclophosphamide together with subcutaneous immunization with racotumomab in alum significantly reduced F3II tumor growth [25]. The antitumor response was comparable to that obtained with standard high-dose chemotherapy in such breast cancer model, but without overt signs of toxicity. Interestingly, combinatory chemo-immunotherapy promoted CD8^+^ lymphocyte tumor infiltration and increased tumor apoptosis [25].

Ganglioside immunotherapies with racotumomab and NGcGM3/VSSP vaccine were well tolerated in animals [24, 38]. In preclinical toxicology studies, the immunization protocol did not affect body weight gain, food and water consumption or induce other signs of overt toxicity in murine models. Subacute toxicity after continuous daily treatment was expressed by an excessive activation of extramedullary myelopoiesis in the spleen and liver in all mice and a strong inflammatory reaction in the lungs, showing dense neutrophil infiltrates in the interalveolar septa [24].

## 4. Expression of NGc in Human Tumors

Tumor-specific expression of NGc-containing gangliosides in some human tumors suggests that the induction of an effective immune response against these antigens may be useful for patients with antigen-positive tumors. The ganglioside NGcGM3 has been described in human neoplasms, including breast carcinoma [33, 34] and melanoma [31], but is usually not detected in normal human cells. This fact defines NGcGM3 as an interesting target for immunotherapy.

As described by Tangvoranuntakul et al. [34] using a monospecific antibody against NGc, staining showed cell type-specific reactivity in adult human tissues. The overall pattern of expression was summarized as prominent in secretory epithelia and associated secretions and present in many blood vessels. In addition, in fetal tissues NGc can be detected in epithelial cells or secretions as well as the placental villus blood vessels [34].

van Cruijsen et al. [35] assessed the possible association of NGcGM3 expression with angiogenesis in lung cancer. They examined 176 samples of nonsmall cell lung cancer (NSCLC) by immunohistochemistry in tissue microarray and found that NGcGM3 is widely expressed in more than 90% of the cases. Microvessel density, as determined by CD34 staining, was lower in NSCLC tissues with high NGcGM3 expression, suggesting that the presence of the ganglioside may favor an antiangiogenic response. Moreover, based on the expression of CD83 which is a marker of mature dendritic cells, NGcGM3 appeared to be involved in tumor-induced dendritic cell suppression [35].

More recently, Scursoni et al. [36] reported for the first time the expression of NGcGM3 in a pediatric solid tumor. They detected the ganglioside in 88% of the cases of Wilms' tumor (nephroblastoma), using the specific anti-NGcGM3 mAb 14F7 and a peroxidase-labeled polymer conjugated to secondary antibodies on postchemotherapy samples. Wilms tumor is considered an ‘‘embryonic tumor” of the kidney, being a mimicry of various elements in normal or abnormal nephrogenesis and presenting a diverse spectrum of histologic appearance. In this regard, Wilms tumor gives the unique opportunity to learn about the expression of NGc gangliosides in diverse transformed cell lineages, comprising epithelial, stromal, and blastemal elements [39]. The strongest expression was found in the epithelial component of Wilms' tumor, and the lower percentage of positive tumor cells was observed in the stromal subtype [36]. Similar results were obtained in a preliminary study with P3, a less-specific mAb that recognizes different NGc-containing gangliosides and sulfatides, including NGcGM3 [40]. More than 70% of Wilms tumors showed a positive staining for NeuGc residues using the P3 antibody [36].

## 5. Immunological Response to NGc in Humans

Targeting ganglioside antigens has been a matter of concern due to the possibility of inducing autoimmune responses. Indeed, in most neuropathies of immunological origin, endogenous gangliosides have been shown to be the target of the autoimmune reactions [41–44]. Antiganglioside antibodies may also affect nonneural tissues, as occuring in systemic lupus erythematosus [45], rheumatoid arthritis, or Sjogren's syndrome [41]. Antibodies reactive to NGc-containing glycolipids have been found to be induced in patients after repeated transfusions with sera from other species [46], in rheumatoid arthritis [47], and also in melanoma patients [32]. Melanoma cells express NGc-containing gangliosides, including NGcGM3 [31], and natural anti-NGc antibodies are increased in melanoma patients. Most interestingly, a significantly higher level of anti-NGc antibodies was demonstrated in those patients who were free of disease more than 5 years after surgery than in those who relapsed within 2 years [32].

In spite of what could be expected, no induction of detrimental autoimmune reactions have been described over decades of clinical development of cancer vaccines targeting endogenous gangliosides such as GM2, GD2, and GD3. In this respect, the heterophilic nature of NGcGM3 is considered an additional asset of this tumor antigen, since, as reviewed in the following sections, the absence of significant expression in normal tissue allows for increased immune responses to immunization while precluding self-targeted reactions.

Breast cancer patients were treated with a regime of 5 biweekly IM injections of NGcGM3/VSSP vaccine followed by monthly boosters. Anti-NGcGM3 IgM and IgG responses were detected in all patients who completed the first 5 injections, collectively termed as “induction phase”. The time course of antibody production showed an overall increase across the 32-week followup, and the maximal recorded titers reached 164,000 for both IgM and IgG. The functional relevance of the induced antibodies was underscored by their capacity to react against an NeuGcGM3^+^ murine tumor cell line and mediated complement-dependent cytotoxicity. Further confirming their specificity for the targeted ganglioside, the induced antibodies reacted as well with human mammary ductal carcinoma cells without staining of surrounding normal tissue [26].

In a second clinical trial, 21 advanced melanoma patients received the same vaccination schedule [30]. Two dose levels were examined: 0.2 and 0.4 mg of ganglioside per dose. The immunogenicity of NGcGM3/VSSP was confirmed, with all evaluable patients eliciting IgM and IgG responses after treatment. Antibody class switching to IgA was detected as well. Delayed-type hypersensitivity (DTH) responses were also evaluated with ID injections of NGcGM3/VSSP. DTH responses were observed in 46% of patients at the 0.2 mg dose level and in 78% of patients at the 0.4 mg dose level. The ganglioside contribution to the specificity of the hypersensitivity reactions was, however, not assessed. No clear relationship could be established between immunological response and the clinical outcome, and the lower dose level was selected for future clinical development due to its safer toxicity profile [30].

The immune response elicited by the murine anti-idiotypic mAb racotumomab was monitored in greater detail and in additional clinical settings. The vaccination regime for racotumomab administration consisted of a 6-dose induction phase followed by monthly boosters. Anti-ganglioside responses were induced in 16/17 melanoma patients [48], 16/16 breast cancer patients, and 16/20 NSCLC patients [27]. The specificity of the induced Ab3 antibodies was assessed by adsorption and analysis of the reactivity of the nonadsorbed fraction. Adsorption with an isotype-matched monoclonal antibody (IgG1) abrogated a small fraction of the racotumomab-directed reactivity, which is an indication of the immunodominance of the racotumomab idiotype over the rest of the IgG1 molecule [27]. Most interestingly, adsorption of Ab3 antibodies with racotumomab preserved 50 to 90% of the reactivity for NGcGM3 in the non-adsorbed fraction, suggesting that the idiotype (Id)^+^/antigen (Ag)^+^ and Id^−^/Ag^+^ specificities are present on separate antibody molecules [48]. Such Id^−^/Ag^+^ antibodies could reflect the activation of an autologous idiotypic cascade in the patient's immune system [27]. The antibodies induced by racotumomab are NGc specific. No cross-reaction was observed to NAcGM3 [48]. The time course of NGcGM3-specific antibodies over 50 weeks in breast cancer patients under racotumomab treatment showed sustained antibody titers. Some patients had detectable ganglioside-specific IgG only by week 30, suggesting that the extended vaccination regime not only is undetrimental to the immune response, but is favorable for late responders as well [29].

As described for NGcGM3/VSSP vaccine, the antibodies induced by racotumomab treatment reacted with an NGcGM3^+^ murine tumor cell line. In addition, racotumomab-induced antibodies were able to react with NGcGM3^+^ lung carcinoma tissue sections [49]. No significant differences were found in the antibody response across the dose levels examined (0.5, 1, and 2 mg). Maximal titers reached about 10,000 in the three dose levels for both IgM and IgG, with no significant differences in the titer means between dose levels [29]. The 1 mg dose level was chosen for further clinical investigation [50].

NGcGM3-specific cellular responses were assessed in racotumomab-treated breast cancer patients. Cryopreserved peripheral blood mononuclear cells were challenged in vitro with ganglioside-loaded, CD1d^+^, autologous monocyte-derived dendritic cells, and the response was measured with an interferon-*γ* immunospot assay. A low frequency of ganglioside-specific interferon-*γ*-secreting cells was detected in 5/13 patients. These cytokine responses were undetectable at baseline and became detectable by weeks 14 to 42, thereby confirming the convenience of an extended vaccination schedule to elicit a ganglioside-specific response [29].

## 6. Toxicity and Preliminary Clinical Outcomes

The main toxicities observed in stage III/IV breast cancer patients receiving the NGcGM3/VSSP vaccine (200 *μ*g per dose) were erythema and induration at the injection site, occasionally associated with mild pain and fever. In spite of the fact that this trial was not adequate for efficacy assessments, a remarkable progression-free survival time was observed in 2 patients with lung metastases [26]. Similarly, in another trial in advanced cutaneous and ocular malignant melanomas, 7 patients treated with the NGcGM3/VSSP vaccine remained alive for more than 2 years after inclusion in the study [30].

In stage III/IV melanoma patients administered with biweekly doses of the anti-idiotype mAb racotumomab (2 mg per dose), the tolerance was satisfactory and no unexpected or serious adverse events were reported. The more frequent adverse event was the local reaction with induration and erythema at the injection site [48]. In a clinical trial in patients with stage III/IV breast cancer, doses of 1 or 2 mg of racotumomab were well tolerated. There were no differences between the two levels of doses tested in toxicity [50]. In another Phase I trial, the toxicity profile of racotumomab was investigated using an extended vaccination protocol of 6 biweekly intradermal injections (induction phase), followed by 10 monthly boosters (maintenance). Nineteen patients with high-risk (stage III) or metastatic breast cancer were vaccinated with different dose levels of 0.5, 1, and 2 mg. Vaccination was relatively well tolerated; local skin reactions grades I and II represented the most common adverse event followed by mild flu-like symptoms lasting for 1 to 2 days. Similar safety results were observed with the 3 tested dose levels [29].

In a compassionate-use basis study, 34 stage IIIb and 37 stage IV NSCLC patients were vaccinated with racotumomab, after receiving standard chemotherapy and radiotherapy [28]. Patients were administered with 5 biweekly injections of 1 mg of racotumomab, other 10 doses at 28-day intervals, and later the patients who maintained a good performance status continued to be immunized at this same time interval. No evidence of unexpected or serious adverse effects was reported. The median survival time of patients, who entered the study with partial response or disease stabilization and with a performance status (PS) 1 after the first line of chemo/radiotherapy, was 11.50 months since the start of vaccination. In contrast, the median survival time calculated for patients who started vaccination with progressive disease and/or a PS2 was 6.50 months [28]. A statistically significant correlation was observed between anti-ganglioside response and survival time in a subset of 20 NSCLC patients from this study. Nonresponder patients (*n* = 4) had a median survival time of 6.35 months (95% CI, 4.97–9.67 months), whereas patients who developed IgG and/or IgM antibodies against NGcGM3 had a median survival time of 14.26 months (95% CI, 5.95–17.3 months; *P* < .01, log rank) [27].

Even though NGcGM3 is a glycolipid, its heterophilic expression allowed a specific immune response when patients were immunized with either a conjugate vaccine (i.e., NGcGM3/VSSP) or an anti-idiotype mAb targeting NGc gangliosides (i.e., racotumomab). Evidence of an NGc-specific cytokine response was observed in some few patients, and its correlation with clinical outcomes remains to be established. The absence of cross-reaction with endogenous gangliosides is inline with the absence of autoimmune toxicity and overall safety profile of both conjugated vaccine and the racotumomab mAb. On the other hand, high titer ganglioside-specific antibody responses were observed in most of the patients, in correlation with survival in a group of NSCLC patients accrued after completion of first-line chemotherapy [27], as described above. The possible involvement of the induced antibodies in a protective antitumor activity is actively being pursued. Recent sequencing and modelling studies suggest that racotumomab might selectively induce Ab3 antibodies with conserved germline sequences specific for heterophilic saccharide antigens [51]. Randomized controlled trials are presently underway and are expected to provide further insight into the role of the induced immune response on the clinical outcome.

In summary, the above-mentioned studies indicate that both vaccines targeted to NGc gangliosides have acceptable safety outcomes and are able to induce specific humoral and cellular immune responses. The response to vaccination seems to be stronger in those patients with lower tumor burden, better performance status, and a good response to previous oncospecific treatment. Also, preliminary evidence suggested that these vaccines may have a positive influence on survival in patients with immune response to NGcGM3 antigen. The current Phase III trials that are being conducted at present will give a definitive answer to the potential clinical benefits offered by these therapeutic vaccines. Furthermore, studies will be required to determine the more efficient combination with chemotherapy or other immune interventions to prevail over the tumor-induced immunosuppression.

## 7. Perspectives

This paper deals with NGc gangliosides—and particularly NGcGM3—as a target for cancer immunotherapy. With the numerous antigens that can be used in immunotherapy, the decision-making process for researchers, hospitals, and companies, in whether or not to invest resources in a specific antigen, has been always a very complicated matter. Fortunately, in a recent work by the National Cancer Institute Translational Research Working Group, Cheever et al. developed a method for prioritization of cancer antigens paving the way to take more rational, informed decisions [7]. Such work aimed to develop a priority-ranked list of cancer vaccine target antigens based on predefined and preweighted objective criteria. An additional aim was testing a new approach for prioritizing translational research opportunities based on an analytic hierarchy process (AHP), a structured technique, and a mathematical model for dealing with complex decisions. Antigen prioritization involved developing a list of “ideal” cancer antigen criteria/characteristics, assigning relative weights to those criteria using pairwise comparisons. The result of the criteria weighting, in descending order, was as follows: (a) therapeutic function, (b) immunogenicity, (c) role of the antigen in oncogenicity, (d) specificity, (e) expression level and percent of antigen-positive cells, (f) stem cell expression, (g) number of patients with antigen-positive cancers, (h) number of antigen epitopes, and (i) cellular location of antigen expression.

Having that work as a reference, we rethought of our experimentation with NGc, and, although is neither on the scope of this paper nor our prerogative to position the antigen in the ranking, we can affirm that NGc somehow matches all of the criteria considered (Table 2), at least in some proportion—as described throughout this paper—whose relative weight should be evaluated by panels of external experts.

Some authors have recently enunciated the introduction of potential biases in the National Cancer Institute Pilot Project [52]. Lang et al. affirmed that the methodology used (AHP) is not well described and is subject to several sources of possible bias, such as participant selection, number of antigens chosen for prioritization, errors in rank order, redundancy, and internal validity. First of all, we differ with Lang et al. in the fact that AHP is not well described, due to being a very well-known technique properly used in a wide variety of settings, including cancer clinical decisions [53, 54]. Cheever et al. clearly described the method by citing the popular work of Bhushan [55] and how AHP is used in a web-based tool [56]. AHP is a powerful tool, used widely in science, and, although it has had some detractors over the years, Forman and Gass. carried out an in-depth paper discussing and rebutting the academic criticisms of AHP [57].

Furthermore, at the time of Cheever's paper publication no cancer vaccine had yet been approved by FDA. However, recent approval of sipuleucel-T for men with advanced prostate cancer, targeting PAP antigen, gave us a valuable lesson on this matter [58]. Interestingly, PAP ranked 26 out of 75 antigens in the ranking of cancer antigen pilot prioritization [7], confirming its capacity to somehow “forecast” those antigens more likely to be translated to patients. Although the ranking is dynamic, given that priorities change as knowledge accrues from new studies, we must reinforce the idea that the associated lists of weighted criteria inform investigators as to what experimental evidence is required to advance antigens to higher priority levels. In this line, those criteria helped us to evaluate that NGcGM3 ganglioside comprised most if not all relevant characteristics as a cancer antigen for vaccine development.

## Figures and Tables

**Table 1:** N-glycosylated (NGc) ganglioside-based cancer vaccines.

Vaccine	Product description	Target antigen	Potential indications	Clinical Phase
Racotumomab	Anti-idiotype murine mAb (1E10 antibody)	NGc-containing gangliosides	Lung cancer Breast cancerPediatric tumors?	Ongoing Phase III
NGcGM3/VSSP	Ganglioside conjugated with bacterial proteoliposomes	NGcGM3 ganglioside	Breast cancer Melanoma Sarcoma?	Ongoing Phase III

**Table 2:** Relevant characteristics of NGcGM3 ganglioside as a cancer antigen, according to the antigen prioritization criteria described by Cheever et al. [7].

Criteria	Data on NGcGM3
Therapeutic function	Clinical data showing that a vaccine-induced clinical responses in at least a small number of patients [26–28]
Immunogenicity	T cell [29] and antibody [26, 27, 29, 30] responses elicited in clinical trials, spontaneous antibody observed in some patients [31, 32]
Oncogenicity	Increased expression in adult [31, 33–35] and pediatric [36] solid tumors, to be determined a clear association with oncogenic process or tissue differentiation
Specificity	Overexpressed in cancer with little or no expression in normal adult tissues [34]
Expression level and % positive cells	Highly expressed on most cancer cells in patients designated for treatment [31, 33, 35, 36]
Stem cell expression	Expression on most cancer cells [35, 36] but without information about putative stem cells
No. of patients with antigen-positive cancers	High level of expression in >80% of patients with a particular tumor type [35, 36]
No. of antigen epitopes	Short antigenic segment with one or few epitopes [31]
Cellular location of antigen expression	Expressed on the cell surface [31, 33, 35, 36] with little or no circulating antigen [10, 11]
